# Multi-focal sequencing of a diffuse intrinsic pontine glioma establishes PTEN loss as an early event

**DOI:** 10.1038/s41698-017-0033-y

**Published:** 2017-09-14

**Authors:** Carl Koschmann, Zishaan Farooqui, Katayoon Kasaian, Xuhong Cao, Daniel Zamler, Stefanie Stallard, Sriram Venneti, Shawn Hervey-Jumper, Hugh Garton, Karin Muraszko, Luigi Franchi, Patricia L. Robertson, Marcia Leonard, Valerie Opipari, Maria G. Castro, Pedro R. Lowenstein, Arul Chinnaiyan, Rajen Mody

**Affiliations:** 1 Department of Pediatrics, Division of Pediatric Hematology/Oncology, Michigan Medicine, Ann Arbor, MI 48109 USA; 2 Department of Pathology, Michigan Medicine, Ann Arbor, MI 48109 USA; 3 Michigan Center for Translational Pathology, Michigan Medicine, Ann Arbor, MI 48109 USA; 4 Comprehensive Cancer Center, Michigan Medicine, Ann Arbor, MI 48109 USA; 5 Howard Hughes Medical Institute, Michigan Medicine, Ann Arbor, MI 48109 USA; 6 Department of Neurosurgery, Michigan Medicine, Ann Arbor, MI 48109 USA; 7 Department of Cell and Developmental Biology, Michigan Medicine, Ann Arbor, MI 48109 USA; 8 Department of Pediatrics, Division of Pediatric Neurology, Michigan Medicine, Ann Arbor, MI 48109 USA

## Abstract

Improved molecular understanding is needed for rational treatment of diffuse intrinsic pontine gliomas (DIPG). Here, using multi-focal paired tumor and germline exome DNA and RNA sequencing, we uncovered phosphatase and tensin homolog (*PTEN*) loss as a clonal mutation in the case of a 6-year-old boy with a diffuse intrinsic pontine glioma, and incorporated copy number alteration analyses to provide a more detailed understanding of clonal evolution in diffuse intrinsic pontine gliomas. As well, using the PedcBioPortal, we found alterations in *PTEN* in 16 of 326 (4.9%) cases of pediatric high-grade glioma (3 of 154 (1.9%) brainstem) for which full sequencing data was available. Our data strengthens the association with *PTEN* loss in diffuse intrinsic pontine gliomas and provides further argument for the inclusion of *PTEN* in future targeted sequencing panels for pediatric diffuse intrinsic pontine gliomas and for the development and optimization of mTOR/PI3K inhibitors with optimal central nervous system penetration.

## Introduction

Brainstem tumors comprise 10–15% of central nervous system tumors in children.^1, 2^ Of these, diffuse intrinsic pontine gliomas (DIPG) are the most common,^1^ and the most aggressive. Despite improvements in diagnostic accuracy and multimodal treatments, prognosis for patients with DIPG remains dismal with a median survival of around 1 year.^3, 4^

Improved molecular understanding is needed for rational treatment of DIPG. Pediatric DIPG has been shown to demonstrate intratumoral spatial histologic heterogeneity.^5, 6^ DIPG appears to be a molecularly homogenous tumor as compared to adult glioblastoma multiforme (GBM).^7, 8^ Deep sequencing of multiple sites within tumors has revealed key mutations, which are inferred to be driving events as they are conserved throughout all samples in tumors with multi-focal analysis. Activating point mutations (H3K27M) in the genes encoding the histone H3 histone family member 3A (*H3F3A*) or histone cluster 1 H3 family member B (*HIST1H3B*) proteins are now understood to be early oncogenic events in DIPG.^7–9^ These mutations are frequently accompanied by driving mutations in genes encoding activin A receptor type 1 (*ACVR1*, one of the bone morphogenetic protein type I receptors), tumor protein p53 (*TP53*), or phosphatidylinositol-4,5-bisphosphate 3-kinase catalytic subunit alpha (*PIK3CA*).^6, 10, 11^ Here, using multi-focal paired tumor and germline exome DNA and RNA sequencing, we uncovered phosphatase and tensin homolog (*PTEN*) loss as a clonal mutation in the case of a 6-year-old boy with DIPG, and incorporated copy number alteration analyses to provide a more detailed understanding of clonal evolution in a DIPG.

## Case report

A 5-year-old boy presented with repetitive episodes of stumbling and tripping, as well as bilateral ptosis. Magnetic resonance imaging (MRI) of the brain revealed a 4.6 cm, diffusely infiltrating pontine mass. Surgical resection was not recommended due to the location and size of the lesion, and he was treated on a Children’s Oncology Group phase I trial (NCT01922076) with AZD1775 (Wee1 kinase inhibitor) concurrently during radiation. Two months following chemo-radiotherapy, the patient underwent convection-enhanced delivery of 8-H9 antibody (NCT01502917). An interval MRI 1 month later was concerning for progressive disease with recurrence of prior symptoms and development of new motor deficits. He was then treated off-trial for six cycles with the histone deacetylase inhibitor panobinostat, based on pre-clinical data showing its efficacy in H3K27M-mutated DIPG.^12^ Approximately 13 months following his initial diagnosis, the patient died from progressive disease. Consent for research autopsy was obtained and research autopsy was performed within hours of patient’s death.

**Fig. 1:**
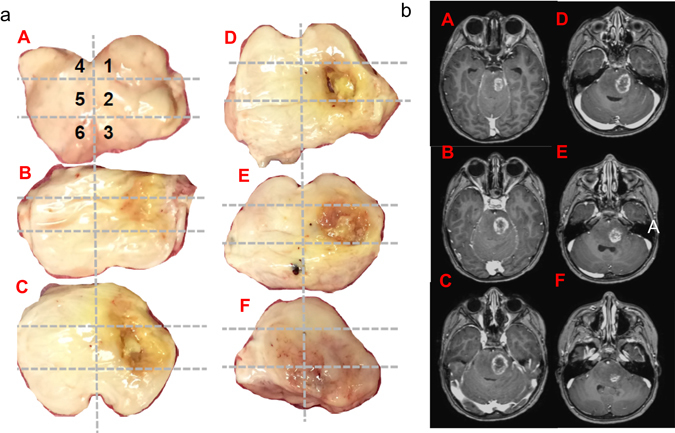
Diffuse intrinsic pontine glioma cross-sections at autopsy and MRI. **a** Histopathological specimens, showing six equal cross-sections of tumor **A**–**F** progressing from superior to inferior sections, with one of six quadrants in each cross-section used for sequencing. **b** Brain MRI (T1 plus contrast) axial images, progressing from superior to inferior sections, demonstrating primarily unilateral necrosis and corresponding contrast enhancement

The involved brainstem was sectioned into six cross-sections (superior to inferior, Fig. 1). Each cross-section was separated into six quadrants, of which one quadrant (e.g., “A1”) from five of the cross-sections was flash frozen and sent for sequencing. Normal brain samples from cerebellum and frontal lobe were also flash frozen and sequenced as germline samples. Five exome libraries and five poly-A transcriptomes were generated from unique flash frozen tumor tissue and one exome and two poly-A transcriptomes were generated from germline samples (normal cerebellum and frontal lobe). Whole exome (paired tumor and germline DNA) and transcriptome (tumor RNA) sequencing was performed on all samples according to previously published methodology in the Michigan Center for Translational Pathology through PEDS-MIONCOSEQ. The PEDS-MIONCOSEQ study was approved by the University of Michigan Institutional Review Board and adheres to the Clinical Laboratory Improvement Amendments.^13^

**Fig. 2:**
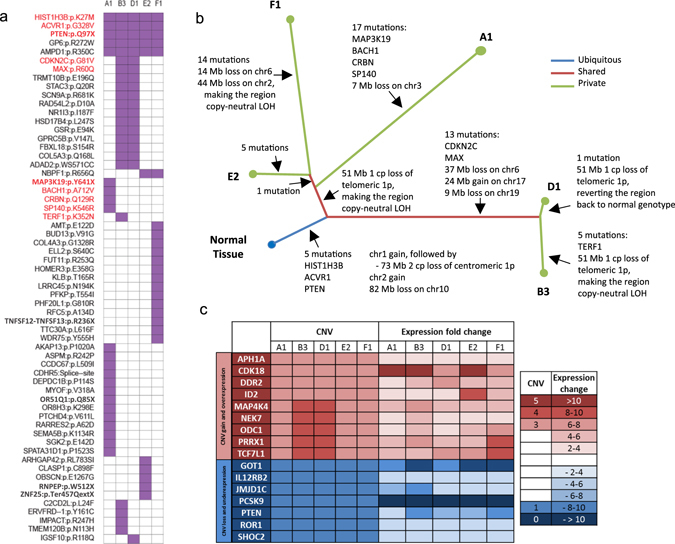
Tumor sequencing and copy number analysis reveal clonal events and tumor evolution. **a** Overall, 61 mutations were noted in the five sequenced tumor samples with five mutations seen in all samples, and previously identified cancer consensus mutations in red (“A1” corresponds to “A1” quadrant in Fig. 1a, etc.) **b** Tumor evolutionary map displays distance corresponding to degree of mutational and copy number alterations. *HIST1H3B*, *ACVR1*, and *PTEN* are conserved in all five specimens. **c** Candidate pathway genes with conserved CNVs and corresponding expression changes by RNA sequencing

Whole exome sequencing revealed 61 somatic mutations distributed throughout the five tumor sites (Fig. 2a), including point mutations in *HIST1H3B* (p.K27M), *ACVR1* (p.G328V), and *PTEN* (p.Q97X), and copy number loss of *PTEN* in all five samples. Somatic mutations were additionally found across all five sites in glycoprotein VI (*GP6*, p.R272W) and adenosine monophosphate deaminase 1 (*AMPD1*, p.R350C), though these mutations are of uncertain significance. We integrated these and the other (sub-clonal) mutations alongside copy number variants (CNVs) (Supplementary Fig. 1), which significantly informed a representative DIPG tumor evolution map (Fig. 2b). Several genes were found to have corresponding CNV and RNA level changes, including nine genes with copy number gain and overexpression and seven genes with copy number loss and reduced expression, including *PTEN* and multiple genes of unclear significance in DIPG pathogenesis (Fig. 2c). Of these genes that exhibited changes in copy number and expression, the most notable alterations in expression were overexpression of cyclin-dependent kinase 18 (*CDK18*), and reduced expression of glutamic-oxaloacetic transaminase 1 (*GOT1*) and proprotein convertase subtilisin/kexin type 9 (*PCSK9)*. While there is no mention of *PCSK9* in the context of oncogenesis in the literature, overexpression of *CDK18* has been seen in association with breast cancer and reduced expression of *GOT1* has recently been demonstrated to be associated with poorer outcomes in glioblastoma.^14–16^ Histopathologic analysis of contralateral quadrants from each cross-section revealed pleomorphic cells infiltrating through the pons. No microvascular proliferation or tumor necrosis was observed and the histology was consistent with a diffuse infiltrative glioma with focal anaplastic changes. Tumor cells showed some expression of glial fibrillary acidic protein. An immunostain for Ki-67 showed marked heterogeneity from region to region (Supplementary Fig. 2).

## Discussion

PTEN is an established negative regulator of PI3K signaling and tumor suppressor in many human cancers.^17–20^ While previously reported in adult high-grade glioma, it has not been regarded as a main driving lesion in pediatric high-grade glioma or DIPG.^10, 21^ Interestingly, one study of DIPG specimens obtained at autopsy utilized comparative genomic hybridization to demonstrate hemizygous loss of chromosome 10q, which includes *PTEN*, in 6 out of 11 samples, as well as one instance of homozygous loss.^6^ From papers with sequencing data, one found hemizygous deletion of *PTEN* in 4 out of 48 (8%) DIPG tumors, and no cases with mutation or homozygous loss; another identified a *PTEN* SNV in 1 of 26 (4%) samples.^22^ Using the PedcBioPortal, which compiled multiple sequencing datasets of human pediatric high-grade glioma and DIPG^10, 22–25^ with additional samples deposited by Dr. Chris Jones (EGAS00001001436), we found alterations in *PTEN* in 16 of 326 (4.9%) cases for which full sequencing is available (3 of 154 (1.9%) brainstem). As well, we found homozygous deletion of *PTEN* in 7 of 834 (0.8%) in which DNA copy number was available (3 of 242 (1.2%) brainstem). The PI3K/mechanistic target of rapamycin (mTOR)/AKT/PTEN pathway is an attractive target for children with DIPG showing *PTEN* loss or *PI3K* activation. The mTOR protein is downstream of PI3K and in vitro data from multiple tumor types with *PIK3CA* mutations demonstrates sensitivity to mTOR inhibition with the mTOR inhibitor everolimus, which exhibits good CNS penetration.^26, 27^ Sapanisertib, an agent that inhibits both mTOR complexes 1 and 2, has been shown to be effective against in vitro and murine models of DIPG.^28^ Recently agents have been explored to dually target PI3K and mTOR, which have shown efficacy in GBM pre-clinically.^29–32^

The prognosis for pediatric DIPG remains bleak despite improved, biopsy-guided multi-modal treatment approaches. It is becoming increasingly evident that a deeper molecular understanding of each individual tumor is needed to guide therapeutic decisions. While our data confirms a relative homogeneity of DIPG in terms of recurrent lesions, our use of copy number analysis and RNA sequencing helps clarify distinct tumor evolution and behavior by region.

In conclusion, we report *PTEN* loss as an early event in whole exome and transcriptome multi-focal sequencing of a case of DIPG. Our data strengthens its association with DIPG, and provides further argument for the inclusion of *PTEN* in future targeted sequencing panels for pediatric DIPG and for the development and optimization of mTOR/PI3K inhibitors with optimal CNS penetration.

### Code availability

Genomic data will be deposited according to established practices of the Michigan Center for Translational Pathology.

## Electronic supplementary material

Supplementary Figure 1. Copy number variant (CNV) and zygosity map for five autopsy sites

Supplementary Figure 2. Histopathology and IHC results for each tumor sample

## Electronic supplementary material

**Supplementary information** accompanies the paper on the *npj Precision Oncology* website (doi:10.1038/s41698-017-0033-y).
