# Supplementary figures and images for: Multi-focal sequencing of a diffuse intrinsic pontine glioma establishes PTEN loss as an early event
Source: NPJ Precis Oncol. 2017 Sep 14;1:32. doi: 10.1038/s41698-017-0033-y (PMC5871904; doi:10.1038/s41698-017-0033-y)

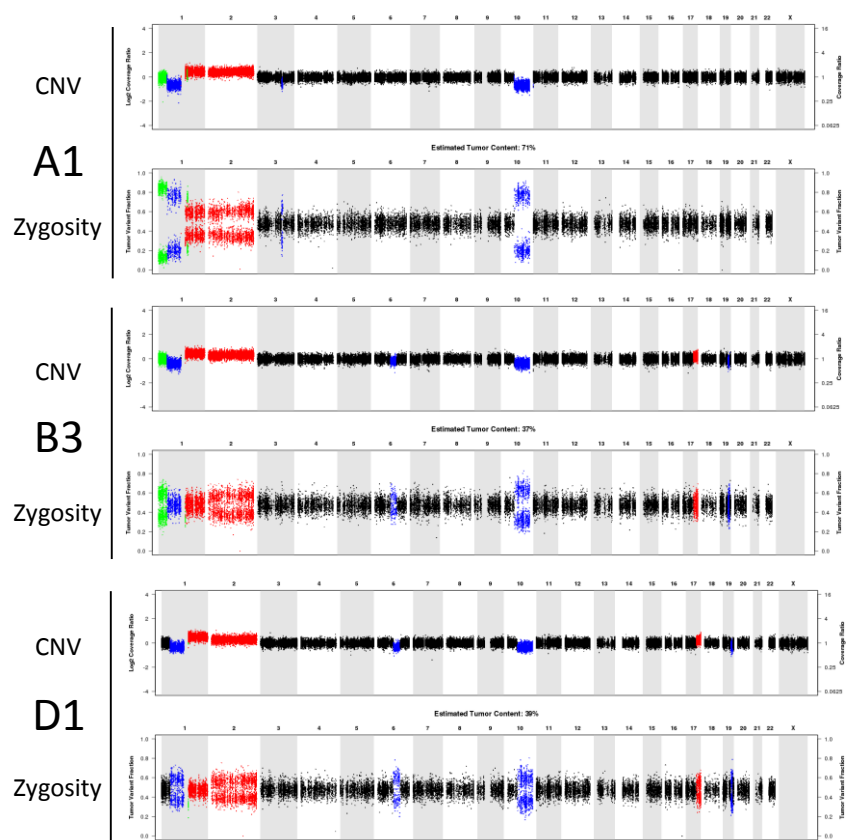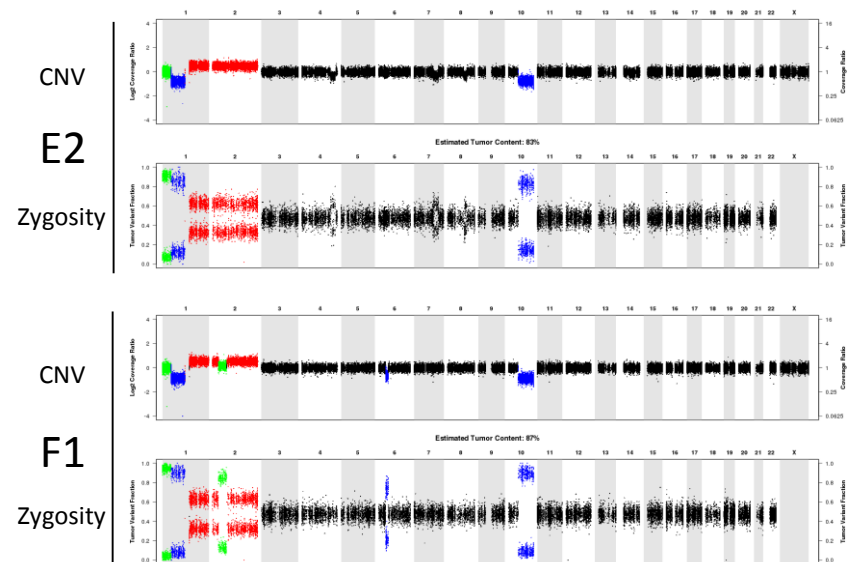

**Supplementary Figure 1. Copy number variant (CNV) and zygosity map for five autopsy sites**

Supplement: Supplementary file 1 — Supplementary Figure 1. Copy number variant (CNV) and zygosity map for five autopsy sites [file 41698_2017_33_MOESM1_ESM.pdf]
