# Supplementary material for: Multi-focal sequencing of a diffuse intrinsic pontine glioma establishes PTEN loss as an early event
Source: NPJ Precis Oncol. 2017 Sep 14;1:32. doi: 10.1038/s41698-017-0033-y (PMC5871904; doi:10.1038/s41698-017-0033-y)
Supplement: Supplementary file 2 — Supplementary Figure 2. Histopathology and IHC results for each tumor sample [file 41698_2017_33_MOESM2_ESM.pdf]

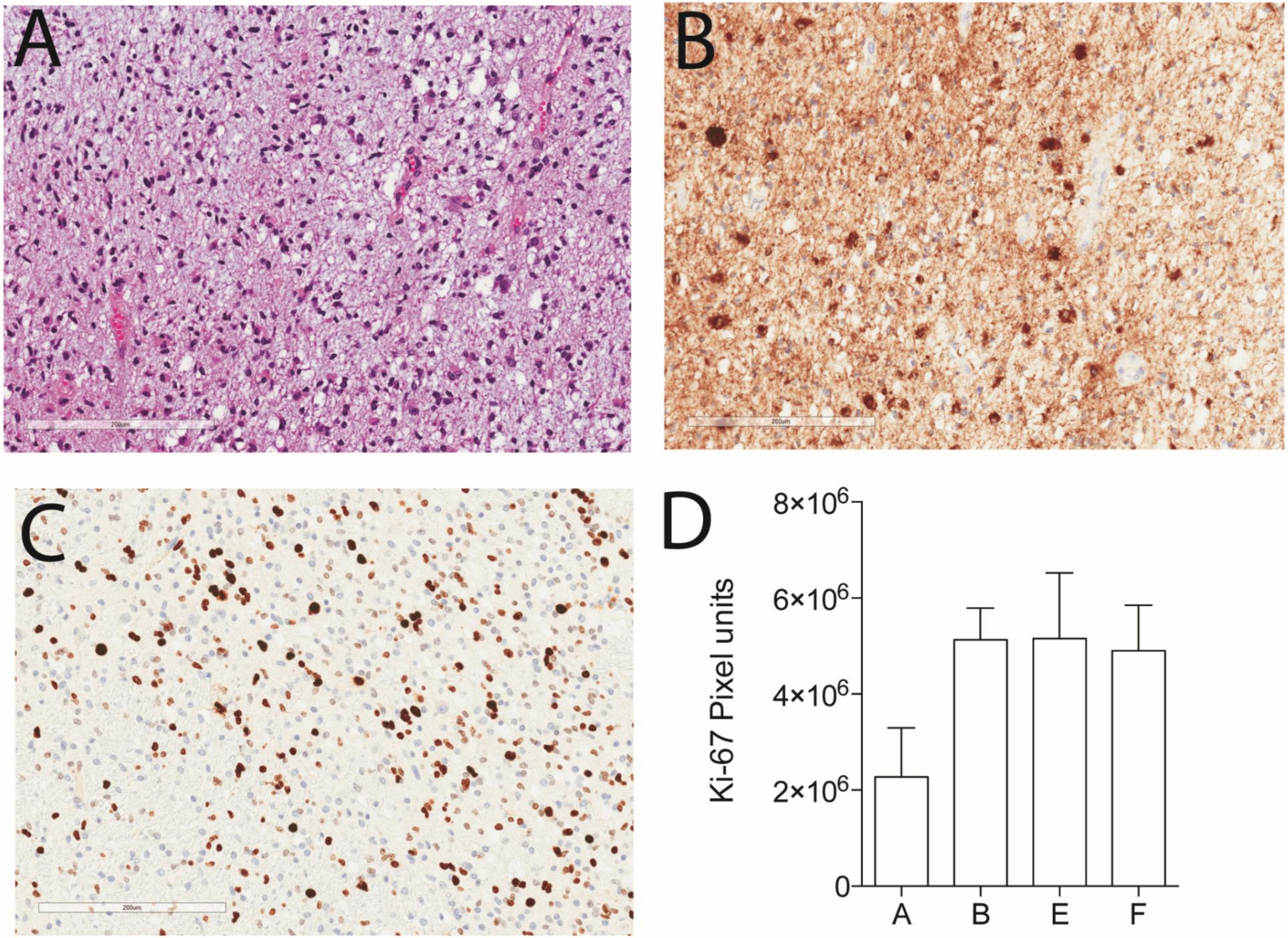

Supplementary Figure 2. Histopathology and IHC results for each tumor sample
(A) Pathology shows a diffuse infiltrative glioma with focal anaplastic changes. (B) Tumor cells show expression of GFAP. (C-D) An immunostain for Ki-67 shows marked heterogeneity from region to region.
